# Distinct immune activation patterns in adult-onset Still’s disease with fungal infections

**DOI:** 10.3389/fimmu.2026.1798932

**Published:** 2026-03-31

**Authors:** Tongxin Wu, Ye Yu, Xuesong Liu, Xiaoxiang Chen, Liyang Gu, Liangjing Lu, Ruru Guo

**Affiliations:** 1 Department of Rheumatology, Renji Hospital, Shanghai Jiao Tong University School of Medicine, Shanghai, China; 2 Department of Ultrasound, Renji Hospital, School of Medicine, Shanghai Jiaotong University, Shanghai, China; 3 Department of Allergy, Renji Hospital, Shanghai Jiao Tong University School of Medicine, Shanghai, China; 4 Shanghai lmmune Therapy Institute, Shanghai, China; 5 Department of Internal Medicine 3-Rheumatology and Immunology, Friedrich-Alexander-Universität Erlangen-Nürnberg and Universitätsklinikum Erlangen, Erlangen, Germany; 6 Deutsches Zentrum Immuntherapie, Friedrich-Alexander-Universität Erlangen-Nürnberg and Universitätsklinikum Erlangen, Erlangen, Germany

**Keywords:** Adult-onset Still’s disease, fungal infection, immune activation, immunobiology, outcome

## Abstract

**Objective:**

Fungal infections are uncommon but potentially life-threatening complications in adult-onset Still’s disease (AOSD). The immunological features of AOSD patients with fungal infections remain poorly defined, and this study aimed to characterize immune activation profiles in this population.

**Methods:**

We retrospectively analyzed 277 patients diagnosed with AOSD, among whom 35 (12.6%) developed fungal infections. Clinical features and immunological parameters, including neutrophil CD64 (nCD64), myeloid-derived suppressor cells (MDSCs), activated T cells, and serum cytokine profiles, were compared between patients with and without fungal infections.

**Results:**

Compared to non-infected AOSD patients, those with fungal infections had a higher frequency of splenomegaly (60.0% vs. 33.5%, p = 0.0024), pulmonary infiltrates (57.1% vs. 20.2%, p < 0.001), and pericarditis (42.9% vs. 20.7%, p = 0.0038). Immunologically, the infection group showed significantly elevated levels of nCD64 (45.39 vs. 11.16, p < 0.05) and enhanced CD8^+^ T cell activation, particularly CD8^+^CD38^+^ T cells (81.54% vs. 66.89%, p < 0.05). Serum soluble IL-2R levels were also higher in infection patients (1884.18 vs. 1259.13 U/mL, p < 0.05). Cytokines such as IL-8, and IL-10 were markedly elevated during infection episodes. Additionally, ferritin elevation remained independently associated with fungal infection after adjusting for disease activity, MAS, and glucocorticoid exposure.

**Conclusions:**

AOSD patients with fungal infections exhibit distinct immune activation patterns involving neutrophils and CD8^+^ T cells, reflecting dynamic immune changes during infection episodes. These immunological features may support integrated immune assessment and inform therapeutic considerations under immunosuppressive treatment.

## Introduction

Adult-onset Still’s disease (AOSD) is a rare systemic inflammatory disorder characterized by high spiking fevers, evanescent rash, arthritis, leukocytosis, and markedly elevated inflammatory markers (1). The onset or relapse of the disease exhibits a certain degree of seasonality. While the precise pathogenesis remains unclear, infectious agents, including viruses (e.g., Epstein-Barr virus, cytomegalovirus), bacteria (e.g., Yersinia, Mycoplasma), and fungi (e.g., Candida spp.) are considered potential triggers in genetically susceptible individuals (2).

Due to frequent use of corticosteroids and immunosuppressants, AOSD patients are highly vulnerable to opportunistic infections. Studies have shown that fungal infections in AOSD (3), though less common than bacterial or viral infections, are associated with disproportionately high morbidity and mortality (4). Commonly reported fungi include Candida spp., Aspergillus, and Pneumocystis jirovecii, affecting sites such as the lungs, bloodstream, and mucosal surfaces (5, 6). Reported case series suggest that fungal infection-related mortality in AOSD patients can exceed 30%, especially in those with delayed diagnosis or severe immunosuppression (6, 7). Therefore, assessing immune status during infection episodes may provide insight into overall immune activation or suppression, thereby supporting adjustments in both antifungal therapy and management of underlying AOSD.

Given the high morbidity and mortality associated with fungal infections in AOSD, there is a clear need to better understand immune dynamics during infection episodes. Beyond pathogen detection, profiling immune parameters provides insight into the patient’s overall immune activation or suppression, which may reflect the combined impact of infection and underlying autoinflammatory disease activity. Our previous work has shown that distinct immunological endotypes in AOSD—defined by differences in innate and adaptive immune cell subsets—are associated with divergent patterns of disease activity, organ involvement, treatment response, and outcome (8, 9). Characterizing immune activation patterns during fungal infection episodes may therefore offer a framework to assess the immune context in which infection and disease activity intersect. In this study, we retrospectively analysed clinical characteristics, outcomes, and immunological profiles of AOSD patients during fungal infection episodes, aiming to delineate distinctive immune activation patterns and their relevance for assessing immune status and underlying disease activity in this high−risk setting.

## Materials and methods

### Study population

We conducted a retrospective study involving 277 patients diagnosed with AOSD at the Rheumatology Department of Renji Hospital between June 2017 and September 2024. All participants met the criteria set forth by Yamaguchi for the diagnosis of AOSD (10). The diagnosis of macrophage activation syndrome (MAS) was based on the HLH-2004 criteria (11). The AOSD patients were divided into two groups: those with fungal infections and those without.

Invasive fungal disease (IFD) was defined according to the revised EORTC/MSG consensus definitions (12, 13). All cases were retrospectively reviewed and classified as proven or probable IFD based on host factors, clinical features and mycological evidence. Proven IFD was defined by the demonstration of fungi in specimens obtained from normally sterile sites by microscopy or culture, or by a positive cryptococcal antigen test in cerebrospinal fluid or blood. Probable IFD required the presence of appropriate host factors, compatible clinical and radiological features, and mycological evidence as defined by the EORTC/MSG criteria. Only patients fulfilling the criteria for proven or probable IFD were included in the final analysis. The fungi identified in this study included Cryptococcus, Candida albicans, Candida glabrata, Candida tropicalis, Pneumocystis jirovecii, and Aspergillus. Patients with documented bacterial or viral infections at admission were excluded. Participants provided informed consent in accordance with the guidelines outlined in the Declaration of Helsinki. The study was approved by the institutional review board of Renji Hospital, Shanghai, China (approval number 2016-083).

### Clinical data collection

We systematically documented demographic information, including age and gender, to establish a comprehensive profile of the cohort. Systemic disease score (range 0–12) was calculated for severity assessment as described by Pouchot et al. (14). Furthermore, we collected pertinent information on baseline treatment regimens, clinical symptoms, and laboratory data directly from the patients’ medical records. All blood samples for routine laboratory tests, immunophenotyping by flow cytometry, and serum cytokine measurements were collected on the day of admission, prior to the initiation or escalation of anti-infective and immunosuppressive treatments; however, in this retrospective study, the exact timing of blood sampling relative to the onset of fungal infection could not be precisely determined. Liver dysfunction was evaluated based on the presence of hepatomegaly and the elevation of liver enzymes, which included aspartate aminotransferase (AST), alanine aminotransferase (ALT), alkaline phosphatase (ALP), and gamma-glutamyl transferase (GGT). To facilitate comparison, the doses of methylprednisolone were converted to their equivalent prednisolone dosages, while doses of other glucocorticoids were standardized accordingly.

### Flow cytometry analysis

Flow cytometry was utilized to evaluate peripheral lymphocyte subsets, neutrophil CD64 (nCD64), T cell activation, and myeloid-derived suppressor cells (MDSCs), with testing carried out by the clinical laboratories at the hospital. Peripheral blood samples were collected in anticoagulated tubes to prevent clotting and were subsequently transported to the laboratory within two hours, maintaining room temperature to preserve cellular integrity. Upon arrival, the samples underwent processing as per the manufacturer’s protocols. Specifically, the blood samples were incubated with the BD Multitest 6-color TBNK reagent. Lymphocyte subsets in the peripheral blood were analyzed using the BD FACSCanto II Flow Cytometer (BD Biosciences, USA), which measured the following populations: CD3+ T cells, CD3+CD4+ T cells, CD3+CD8+ T cells, CD19+ B cells, and CD3−CD16+CD56+ NK cells. T cell activation was assessed by identifying CD3+HLA-DR+ T cells, CD4+HLA-DR+ T cells, CD8+HLA-DR+ T cells, CD4+CD38+ T cells, and CD8+CD38+ T cells. For MDSCs, total MDSCs were classified as CD45+HLA-DR−CD33+CD11b+ cells, whereas polymorphonuclear MDSCs (PMN-MDSCs) and monocytic MDSCs (M-MDSCs) were identified as CD15+ and CD14+ respectively.

### Detection of serum cytokines

Serum cytokine levels were quantified using a cytometric bead array (CBA) from Cellgene Biotech, following the manufacturer’s instructions. This multi-cytokine detection kit is designed to accurately measure a diverse array of cytokines, including IL-1β, IL-2, IL-4, IL-5, IL-6, IL-8, IL-10, IL-12p70, IL-17A, TNF-α, IFN-α, and IFN-γ. In brief, the obtained serum was then incubated with a combination of cytokine capture beads and detection beads. The capture beads are coated with specific antibodies that bind to the target cytokines present in the serum, while the detection beads are conjugated with fluorescent labels that allow for subsequent quantification. Samples were analyzed on the BD FACSCanto II Flow Cytometer (BD Biosciences, USA), and the resulting data were processed using FCAP Array software version 3.0 (BD Biosciences).

### Statistical analysis

Continuous variables were presented as either median with interquartile range (IQR) or mean ± standard deviation (SD), while categorical variables were reported as count (percentage). To assess differences between the two groups, continuous variables were analyzed using the Student’s t-test or Mann–Whitney U test, depending on the data distribution. Categorical data were evaluated using the chi-square test or Fisher’s exact test, as appropriate. To control for multiple testing, P values for immunological and cytokine comparisons were adjusted using the Benjamini–Hochberg false discovery rate (FDR) procedure. Correlation analyses were conducted using Spearman’s rank correlation coefficient. Multivariable linear and logistic regression models were used to assess the associations of infection with continuous outcomes (ferritin, IL−6) and the binary outcome (MAS), adjusting for age, Pouchot score, and glucocorticoid dose. A two-tailed P value of less than 0.05 was deemed statistically significant. Data analyses were executed using SPSS version 26.0 and R (version 4.5.1).

## Results

### Demographic and clinical characteristics of AOSD patients

A total of 277 patients with AOSD were included in the study, among which 35 (12.6%) were diagnosed with fungal infections. The majority of these infections were located in the respiratory tract, affecting 26 patients, which accounted for 74.3% of the infection cases. Baseline characteristics are detailed in **Table 1**. The age of patients in the infection group was significantly greater than that in the non-infection group, with a mean age of 47.14 years compared to 40.59 years (p = 0.0374). Additionally, patients with infections exhibited a higher Pouchot score (6.54 vs. 5.34, p < 0.001) than their non-infected counterparts.

**Table 1:** Baseline characteristics and therapy of AOSD patients.

Variable	Non-infection (n=242)	Infection (n=35)	P-value
Age (years)	40.59 ± 15.92	47.14 ± 17.02	0.0374
Male sex, n (%)	55 (22.7)	12 (34.3)	0.1356
Pouchot score	5.34 ± 1.74	6.54 ± 1.56	<0.001
WBC>15 x 10^9/L (%)	85 (35.1)	8 (22.9)	0.1509
MAS, n (%)	31 (12.8)	22 (62.9)	<0.001
Medication			
Prednisolone (n, %)	226 (93.4)	22 (62.9)	<0.001
Maximal dose (mg/day), Median (IQR)	40.00 (30.00,60.00)	40.00 (0.00,60.00)	0.0625
Maximal dose (mg/day), Median (IQR)	40.00 (0.00,80.00)	40.00 (0.00,80.00)	0.5816
Intravenous dexamethasone therapy (n, %)	92 (38.0)	27 (77.1)	<0.001
Maximal dose (mg/day), Median (IQR)	0.00 (0.00,15.63)	20.00 (10.00,30.00)	<0.001
Maximal glucocorticoid dose (mg/day), Median (IQR)	75.00 (50.00,133.33)	133.33 (100.00,200.00)	<0.001
MTX, n (%)	91 (37.6)	3 (8.6)	<0.001
CsA, n (%)	61 (25.2)	12 (34.3)	0.2545
Tacrolimus, n (%)	15 (6.2)	1 (2.9)	0.6859
Intravenous immunoglobulin, n (%)	28 (11.6)	10 (28.6)	0.0135
VP-16, n (%)	20 (8.3)	9 (25.7)	0.0043
Anti-IL-6R, n (%)	24 (9.9)	3 (8.6)	0.8019

AOSD, Adult-onset Still’s disease; MAS, macrophage activation syndrome; MTX, Methotrexate; CsA, Cyclosporine A.

**Figure 1:**
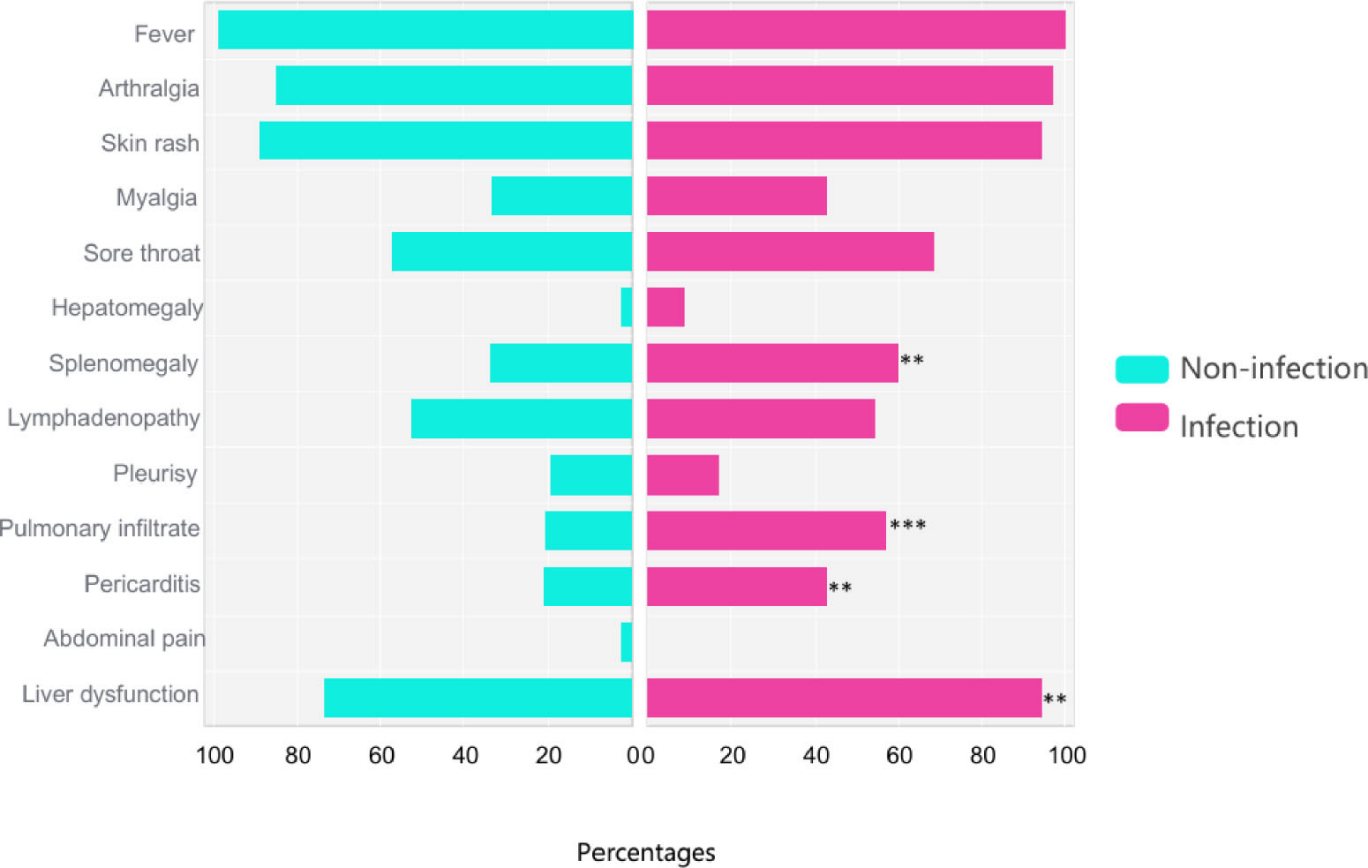
Comparisons of clinical manifestations between AOSD patients without and with infection. *p < 0.05, **p < 0.01, ***p < 0.001.

During their hospital stay, 22 out of the 35 infected patients (62.9%) developed macrophage activation syndrome (MAS), which is a significantly higher proportion compared to 31 out of 242 (12.8%) in the non-infection group (p < 0.001). We performed multivariable logistic regression in the full cohort to identify predictors of MAS. Infection emerged as the strongest predictor (OR 8.00, 95% CI 3.07–22.41, P < 0.001), together with Pouchot score and steroid dose (**Supplementary Table 1**). Furthermore, the maximum glucocorticoid dosage was notably greater in the infection group (133.33 mg/day) than in the non-infection group (75.00 mg/day, p < 0.001). The incidences of splenomegaly (33.5% vs. 60.0%, p = 0.0024), pulmonary infiltrates (20.2% vs. 57.1%, p < 0.001), and pericarditis (20.7% vs. 42.9%, p = 0.0038) were significantly lower in AOSD patients without fungal infections compared to those with infections. Additionally, liver dysfunction was observed in 33 out of 35 (94.3%) patients in the infection group, which was significantly higher than the 177 out of 242 patients (73.1%) in the non-infection group (p = 0.0063) (**Figure 1**).

### Alterations of immunologic and laboratory findings during infection

**Figure 2:**
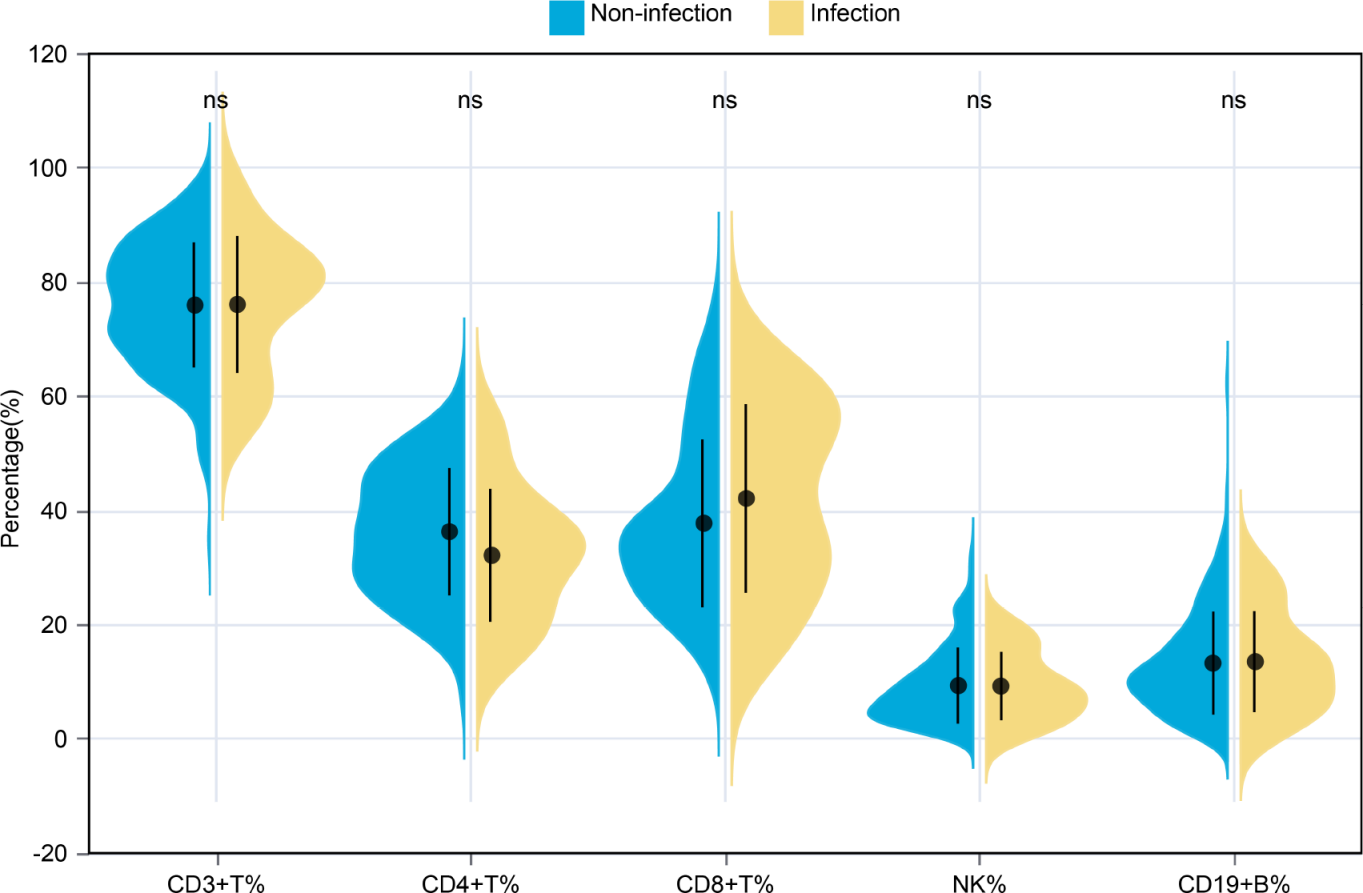
Comparisons of lymphocyte subsets percentages between the infection and non-infection groups. ns, p > 0.05.

We examined the changes in immunological and laboratory parameters during the course of infection. The infection group exhibited a lower percentage of lymphocytes (5.90% vs. 9.95%, p = 0.0332) compared to the non-infection group. Additionally, the level of C3 was significantly reduced in the infection group (1.14 g/L vs. 1.32 g/L, p = 0.0030). Patients with infections showed markedly elevated levels of ferritin (8864.38 ng/mL vs. 2271.32 ng/mL, p < 0.001), alanine aminotransferase (ALT) (72.00 U/L vs. 38.00 U/L, p = 0.0051), and aspartate aminotransferase (AST) (49.00 U/L vs. 33.80 U/L, p = 0.0046) (shown in **Table 2**). However, no significant differences were noted in the proportions of lymphocyte subsets, including CD3+, CD4+, and CD8+ T cells, CD19+ B cells, and CD3−CD16+CD56+ NK cells (**Figure 2**).

**Table 2:** Immunologic and laboratory findings in AOSD patients.

Variable	Non-infection(n=242)	Infection(n=35)	P-value
WBC (x10^9/L)	13.28 ± 7.51	11.23 ± 7.35	0.1299
Neu (%)	81.07 ± 13.44	82.10 ± 18.01	0.6857
Neu(x10^9/L)	11.37 ± 7.47	9.80 ± 7.15	0.2431
Lym(x10^9/L)	1.27 ± 0.79	0.93 ± 0.89	0.0172
Mon (x10^9/L)	0.50 ± 0.27	0.45 ± 0.35	0.2869
Lym%	9.95 (5.98,16.15)	5.90 (3.70,13.80)	0.0332
B%	13.25 ± 9.00	13.50 ± 8.82	0.8812
C3(g/L)	1.32 ± 0.30	1.14 ± 0.30	0.0030
C4(g/L)	0.30 ± 0.14	0.29 ± 0.14	0.8226
Ferritin(ng/mL)	2271.32 ± 8266.92	8864.38 ± 10113.39	<0.001
Hemoglobin(g/L)	110.93 ± 18.51	100.54 ± 22.56	0.0028
Platelet(x10^9/L)	275.28 ± 129.30	169.37 ± 114.35	<0.001
ALT(U/L)	38.00 (21.00,86.00)	72.00 (43.00,107.00)	0.0051
AST(U/L)	33.80 (19.00,52.90)	49.00 (30.00,119.00)	0.0046
LDH(U/L)	533.49 ± 649.34	687.71 ± 565.56	0.1461
Triglycerides (mmol/L)	1.76 ± 1.07	2.32 ± 1.41	0.0088
Fibrinogen (g/L)	3.96 ± 1.86	3.66 ± 2.01	0.3702
CRP (mg/L)	38.25 (11.68,78.23)	45.66 (8.42,106.56)	0.4898
ESR (mm/H)	51.29 ± 32.15	44.91 ± 28.91	0.2748

WBC, White blood cells; NEU, Neutrophil; Lym, Lymphocyte; Mon, Monocyte; ALT, Alanine aminotransferase; AST, Aspartate aminotransferase; LDH, Lactate Dehydrogenase; CRP, C-reactive protein; ESR, erythrocyte sedimentation rate.

### Activation of innate and adaptive immune cells

**Figure 3:**
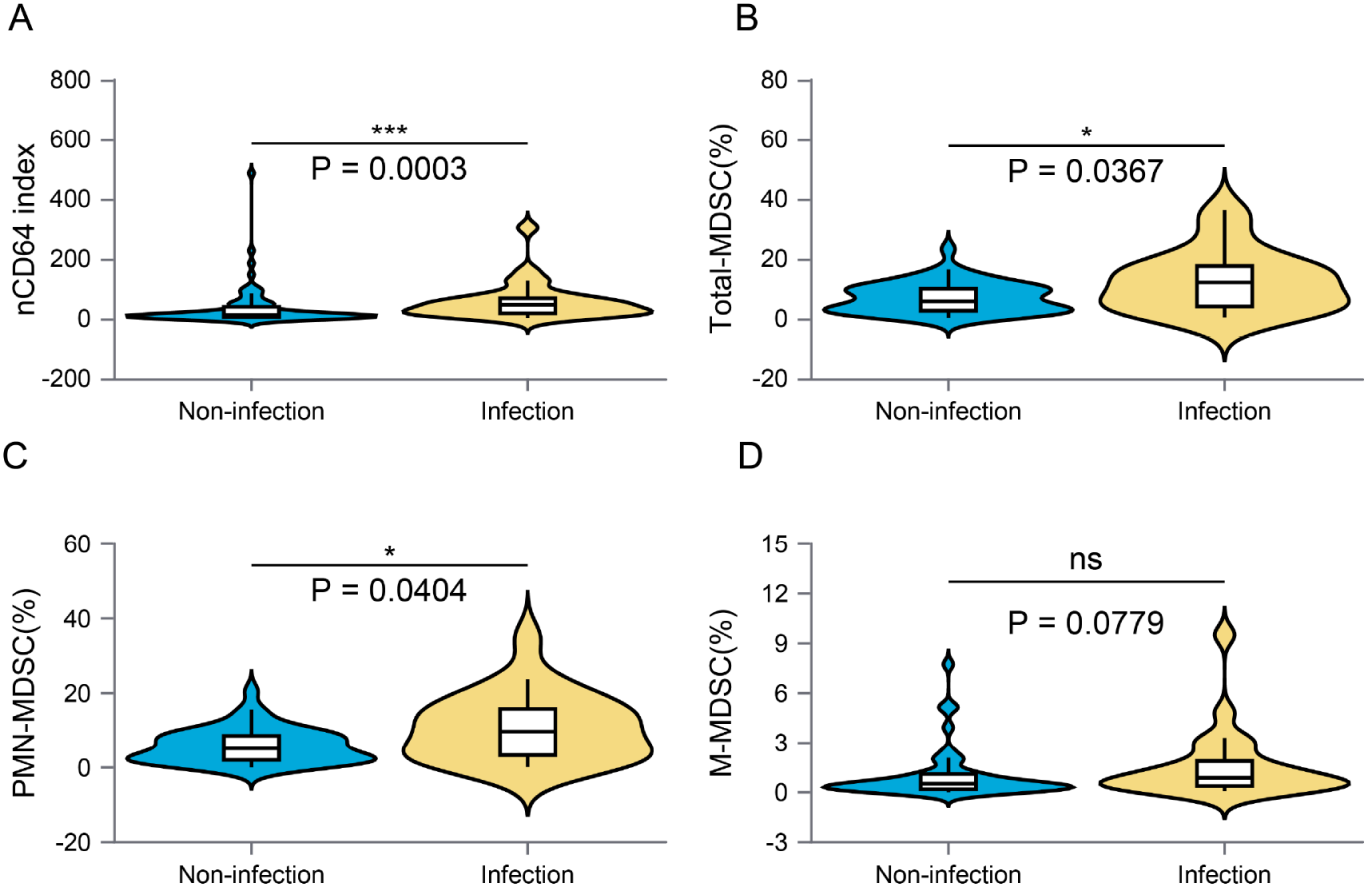
Activation of innate immune cells was compared between the non-infection and infection groups. **(A)** presents the nCD64 values, **(B–D)** illustrate the proportions of total MDSCs, PMN-MDSCs, and M-MDSCs. MDSCs refer to myeloid-derived suppressor cells, with PMN-MDSCs denoting polymorphonuclear myeloid-derived suppressor cells and M-MDSCs indicating monocytic myeloid-derived suppressor cells. P values shown are unadjusted; none of the MDSC-related comparisons remained significant after FDR correction. ns, p > 0.05, *p < 0.05, **p < 0.01, ***p < 0.001.

We conducted a further analysis to evaluate the activation status of immune cells during infection in these immunocompromised patients. The infection group exhibited a significantly elevated level of nCD64 (45.39 vs. 11.16, p < 0.001) (**Figure 3A**). Additionally, myeloid-derived suppressor cells (MDSCs) were found in higher proportions among AOSD patients with infections compared to those without (12.35% vs. 7.21%, unadjusted p = 0.0367) (**Figure 3B**). The two major subsets, PMN-MDSCs and M-MDSCs, also showed numerically higher frequencies in the infected patients (10.69% vs. 6.20%, p = 0.0404, and 0.87% vs. 0.52%, unadjusted p = 0.0779, respectively) (**Figures 3C, D**). However, these differences, including total MDSCs as well as PMN-MDSCs and M-MDSCs, did not remain statistically significant after false discovery rate correction, likely due in part to the limited sample size and reduced statistical power (**Supplementary Table 2**).

**Figure 4:**
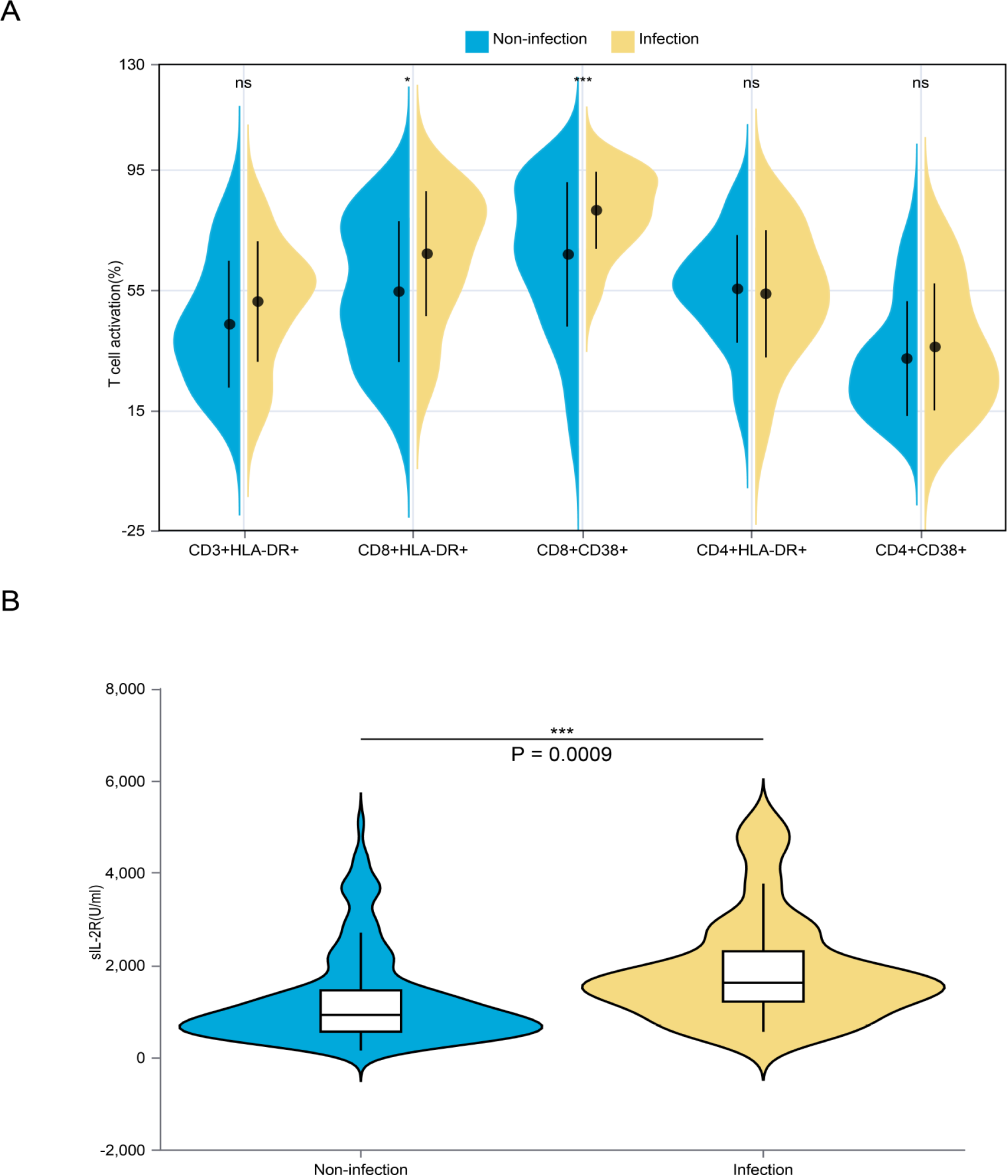
Comparisons of activation of adaptive immune cells between the non-infection and infection groups. **(A)** Expression of activation markers on CD3+ T cells, CD4+ T cells and CD8+ T cells. **(B)** serum levels of IL-2 receptor in both groups. ns, p > 0.05, *p < 0.05, ***p < 0.001.

The immunological status of adaptive immune cells was analyzed with a particular emphasis on T cells. During the infection, no significant changes were observed in the proportions of CD3+HLA-DR+ T cells, CD4+HLA-DR+ T cells, and CD4+CD38+ T cells (**Figure 4A**). The proportion of CD8^+^CD38^+^ T cells was significantly higher in patients with fungal infections compared with those without (81.54% vs. 66.89%, p < 0.05) and remained significant after false discovery rate correction. By contrast, although CD8^+^HLA-DR^+^ T cells were higher in the infection group (67.12% vs. 54.91%), this difference did not retain statistical significance after multiple-testing adjustment, but showed a non-significant trend (p = 0.0641) (**Supplementary Table 2**).Additionally, we evaluated the serum levels of soluble IL-2R (sIL-2R), a biomarker that is thought to be released during T cell activation. The result demonstrated a significantly elevated level of IL-2R in the infection group (1884.18 U/ml vs. 1259.13 U/ml, p < 0.001) (**Figure 4B**).

### Expression of cytokines during immune response fluctuations

**Figure 5:**
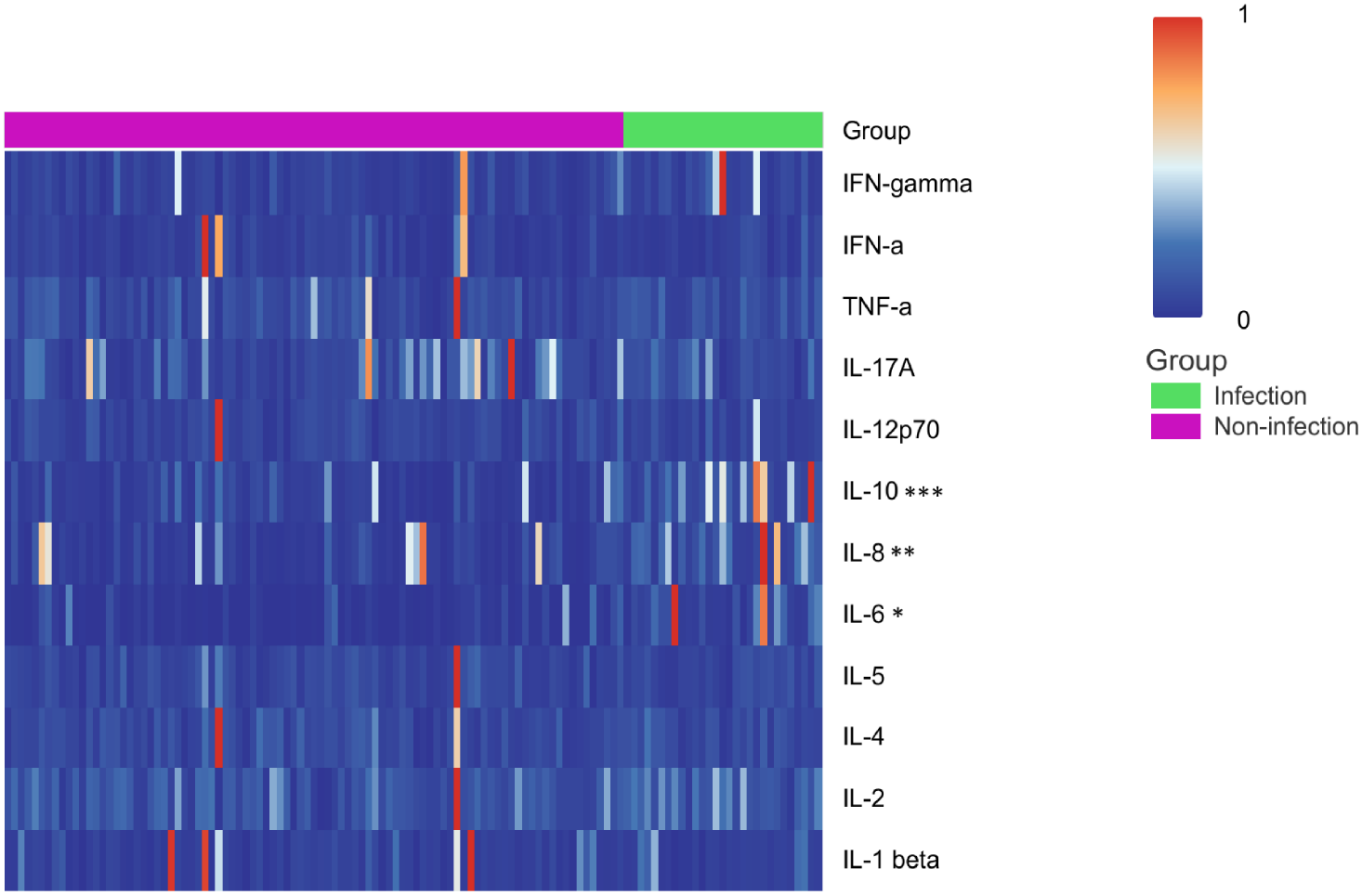
Heatmap illustrated levels of serum cytokines in the non-infection and infection groups, with each group clearly labeled at the top. The data presented in the heatmap is color-coded according to the normalized values for each cytokine, where blue signifies low levels and red indicates high levels. *p < 0.05, **p < 0.01, ***p < 0.001.

The overall profile of serum cytokines can reflect the status and functionality of immune cells. We conducted a comparison of 12 different cytokines in serum samples from the infection group (n = 29) and the non-infection group (n = 91). The analysis revealed significantly elevated levels of IL-6, IL-8, and IL-10 in the infection group, while IL-17A did not reach statistical significance as depicted in the heatmap (21.26 pg/ml vs. 7.63 pg/ml, p = 0.0210; 20.38 pg/ml vs. 13.02 pg/ml, p = 0.0050; 10.99 pg/ml vs. 5.63 pg/ml, p < 0.001, respectively, **Figure 5**). After FDR correction, sIL-2R, IL-8 and IL-10 remained significantly different between the two groups, whereas the other parameters, including IL-6, did not retain statistical significance (**Supplementary Table 2**).

To evaluate whether the observed elevations in CD8+HLA-DR+ T cells, ferritin and IL−6 were independent of disease activity, MAS, and glucocorticoid exposure, multivariable linear regression was performed, indicating that fungal infection was independently associated with higher ferritin levels, after adjusting for Pouchot score, steroid dose, and age (β = 0.78, 95% CI 0.20–1.37, P = 0.009). Sensitivity analysis excluding MAS from the model yielded consistent results, confirming that the association was not solely driven by MAS (**Supplementary Table 3**; **Supplementary Figure 1**).

## Discussion

There has been significant interest in the possible pathogenetic roles of various bacterial and viral agents in AOSD (2), with some evidence suggesting that viral exposures may influence disease onset (15). In this study, we observed that the proportions of both innate and adaptive immune cells in peripheral blood displayed complex fluctuations during fungal infections and fungal infection was an independent risk factor for MAS in patients with AOSD. The results underscore the importance of early identification and management of infections to mitigate MAS risk. Notably, infected patients exhibited diminished levels of circulating lymphocytes, likely due to pathogen-induced cell death and the inhibition of lymphocyte proliferation (16, 17). However, it is also possible that lymphocyte depletion caused by exposure to immunosuppressants in AOSD patients leads to reduced immunity, thereby increasing the risk of opportunistic infections (6, 7). These findings highlight the complex immune landscape during fungal infections.

The myeloid cells present in the blood of infected patients with AOSD exhibited significant alterations in their activation phenotypes. Normally, CD64 (FcγRI) is infrequently expressed on neutrophils; however, its levels rise significantly during infections (18). Consistent with various studies focused on sepsis and infections (19–21), our research identified elevated levels of nCD64 in the fungal infection group. Activated neutrophils are essential components of the innate immune response, playing a critical role in defending against bacterial, fungal, and even viral infections (22). In AOSD patients with fungal infections, nCD64 levels were higher compared with previously reported infection cohorts (23), suggesting a greater likelihood of both infection and underlying disease activity. Myeloid-derived suppressor cells (MDSCs), consisting primarily of two subsets—PMN-MDSCs and M-MDSCs—are pathologically activated myeloid cells with immunosuppressive functions (24–26). We observed a trend toward increased circulating MDSC frequencies in AOSD patients with fungal infections; however, their functional properties and mechanistic roles could not be determined due to the phenotypic nature of our analysis.

Concerning adaptive immune cells, we found that patients with concurrent fungal infections had decreased absolute lymphocyte counts. Further, we evaluated lymphocytes activation status. Our study found an increase in the subpopulations of CD8+ CD38+ T cells among patients with infections. These activated CD8+T cells may contribute to antimicrobial immune responses by facilitating the clearance of infected cells, but also promote a broader inflammatory milieu through increased cytokine production (27, 28), which could potentially modulate the activity of the underlying AOSD.

Cytokines are key mediators mainly released by activated immune cells and play central roles in coordinating innate and adaptive immune responses. In our study, AOSD patients with fungal infections exhibited increased serum levels of IL-8 and the anti-inflammatory cytokine IL-10, both of which remained significant after FDR correction, whereas the difference in IL-6 did not retain statistical significance after multiple-testing adjustment. IL-8 is a chemoattractant for neutrophils and contributes to the amplification of inflammatory responses at sites of infection (29). IL-10 may exert immunoregulatory effects, limiting excessive inflammation and tissue damage during infections (30). Although IL-17 is important for antifungal immunity (30, 31), its plasma levels were not significantly different in patients with concomitant fungal infection, suggesting that local immune responses at the infection site warrant further investigation. Collectively, these findings, together with the results after FDR correction, indicate that inflammatory responses during episodes of concomitant fungal infection largely overlap with underlying AOSD-related immune activity, resulting in complex and dynamic immune fluctuations rather than representing an infection-specific immune signature.

This study has several limitations. First, its retrospective design limited immune assessments to admission samples, preventing evaluation of temporal dynamics before and after fungal infection or antifungal therapy. Second, as a single-centre study in China with a small number of fungal cases, selection bias may exist and generalisability is limited. Third, although only proven or probable invasive fungal infections were included, mixed or sequential infections during hospitalization could not be fully excluded, potentially affecting observed immune profiles. Fourth, Comorbidities (e.g., diabetes) and recent viral infections, known to influence immunity (31), were not systematically recorded. Despite these limitations, this study provides the first characterization of immune perturbations in AOSD patients with fungal infections, highlighting the need for early vigilance and timely immune assessment in this high-risk population.

Taken together, our study provides a comprehensive overview of systemic immune alterations in AOSD patients with fungal infections. Infected patients showed higher disease activity, more organ involvement, and increased MAS incidence, accompanied by MDSC expansion, CD8^+^ T cell and neutrophil activation, and elevated pro-inflammatory and regulatory cytokines. While these responses support antifungal defense, their dysregulation may aggravate autoinflammation and disease progression, underscoring the need for close immune monitoring in this population.

## Data Availability

The original contributions presented in the study are included in the article/**Supplementary Material**. Further inquiries can be directed to the corresponding authors.
