# Supplementary material for: Distinct immune activation patterns in adult-onset Still’s disease with fungal infections
Source: Front Immunol. 2026 Mar 31;17:1798932. doi: 10.3389/fimmu.2026.1798932 (PMC13076156; doi:10.3389/fimmu.2026.1798932)

Supplementary material

AOSD Multivariable Regression Results

Supplementary Table 1. Multivariable logistic regression for MAS

Variable	OR	Lower 95% CI	Upper 95% CI	P value
infection	8.001	3.065	22.405	0.00003650
age	0.993	0.967	1.017	0.55500000
pouchot	2.037	1.551	2.767	0.00000121
steroid	1.009	1.004	1.015	0.00036700

Supplementary Table 2. Immunological Parameters Comparison after FDR

Marker	FDR	Infection (Mean)	non-infection (Mean)
**nCD64**	**0.00465**	57.256	34.173
Total_MDSC	0.24900	12.351	9.735
PMN_MDSC	0.24900	10.686	8.658
M_MDSC	0.17300	1.617	1.026
CD3_HLA_DR	0.17300	51.248	44.038
CD8_HLA_DR	0.06410	67.119	55.004
**CD8_CD38**	**0.02990**	81.541	67.122
CD4_HLA_DR	0.79900	53.812	55.472
CD4_CD38	0.56700	36.169	32.708
**sIL_2R**	**0.00647**	1,884.177	1,259.128
IL1b	0.17300	3.034	5.540
IL2	0.13900	2.265	4.158
IL4	0.79900	1.847	2.835
IL5	0.65600	1.120	1.701
IL_6	0.13800	29.087	50.279
**IL_8**	**0.02990**	45.598	65.323
**IL_10**	**0.00465**	21.340	19.528
il12p70	0.14600	1.796	2.128
il17a	0.79900	3.795	11.631
TNFa	0.68300	2.824	4.933
IFNα	0.52900	2.185	3.877
IFNγ	0.17800	6.447	6.194

Supplementary table 3. Multivariable linear regression of log(ferritin)

Variable	estimate	CI	p.value
(Intercept)	3.86	[2.87, 4.86]	0.00000000000123
infection	0.78	[0.2, 1.37]	0.00918000000000
pouchot	0.36	[0.23, 0.5]	0.00000044600000
steroid	0.00	[0, 0.01]	0.00275000000000
age	0.02	[0, 0.03]	0.00830000000000

Supplementary Figure 1. Forest plot of regression estimates across biomarkers

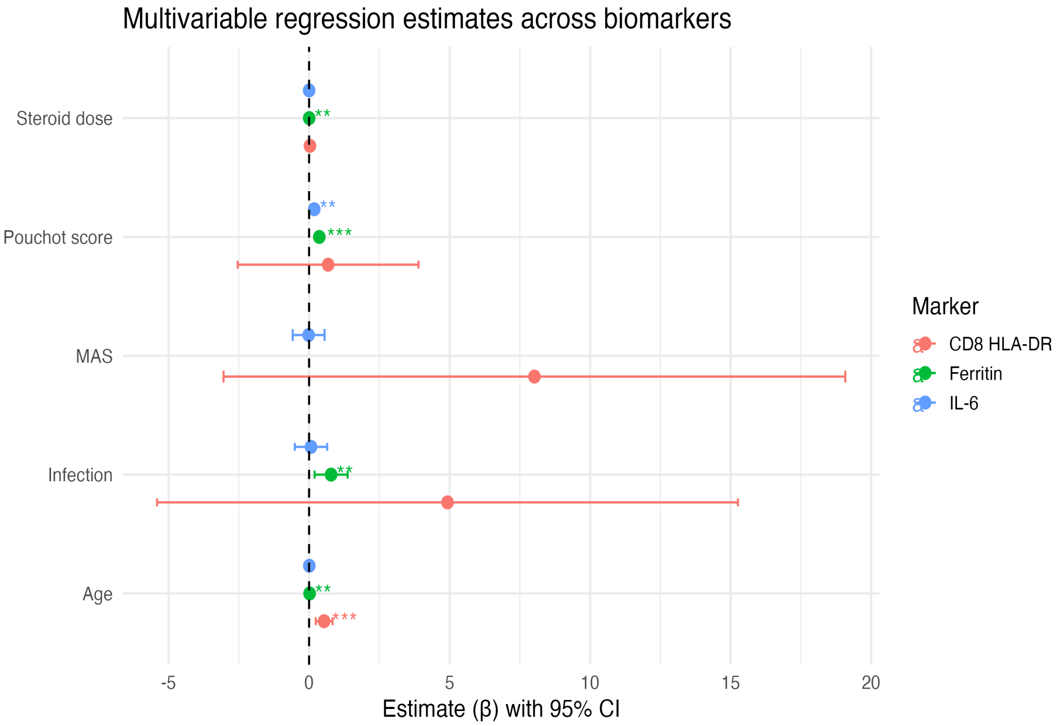

Supplement: Supplementary file 1 [file DataSheet1.pdf]
